# Post-Viral Olfactory Loss: What We Learned from the SARS-CoV-2 Pandemic

**DOI:** 10.3390/life12111868

**Published:** 2022-11-12

**Authors:** Luigi Angelo Vaira, Giovanna Deiana, Fabio Maglitto, Giovanni Salzano

**Affiliations:** 1 Maxillofacial Surgery Operative Unit, Department of Medicine, Surgery and Pharmacy, University of Sassari, 07100 Sassari, Italy; 2 Biomedical Science Department, PhD School of Biomedical Science, University of Sassari, 07100 Sassari, Italy; 3 Direction, Hygiene and Hospital Infection Control Operative Unit, University Hospital of Sassari, 07100 Sassari, Italy; 4 Maxillofacial Surgery Operative Unit, University Hospital of Naples “Federico II”, 80131 Naples, Italy

Viral infections have always been one of the most frequent causes of persistent olfactory dysfunctions accounting for 18% to 45% of all cases [1,2]. However, the exact prevalence of olfactory dysfunctions during common flu has never been determined with absolute certainty; the risk factors for the development of persistent disorders are unknown and there are no therapeutic guidelines. The management of patients with olfactory disorders was the prerogative of a few smell specialists and is mostly unknown to the rest of the healthcare community.

The COVID-19 pandemic represented a marked turning point in this field. First, it caused a large number of people to lose their sense of smell. The prevalence of olfactory dysfunctions in the acute phase of the infection was greater than 50% in the first pandemic waves [3,4,5,6] and continues to remain significant, even for the Omicron variant [7,8]. Moreover, the great media coverage that olfactory disorders have received in recent years has contributed to raising the awareness of this type of problem among the public. The number of patients requesting assistance for olfactory disorders of various etiology is therefore constantly increasing and it is no longer possible, as often happened in the past, not to offer them solutions or ignore their requests [9,10].

SARS-CoV-2 has demonstrated the ability to induce persistent severe olfactory disturbances in approximately 5% of all those infected [11,12,13]. A huge number of individuals experience severe long-term morbidity, with devastating effects on quality of life, which can lead to social isolation or exposure to environmental dangers [14,15]. On the other hand, the pandemic has pushed research in this area and uncovered a huge amount of information on post-viral olfactory disorders. In the past, this has not been possible because patients sought assistance at a great distance from the infection, when it was no longer possible to identify the pathogen [2]. Moreover, the psychophysical tests with which the olfactory function is assessed must be administered in person, introducing safety problems for operators who dispute their use to evaluate contagious patients. During the pandemic, many researchers bravely evaluated patients within isolation departments, and for the first time, we were able to estimate the prevalence of olfactory dysfunctions during an infection [16,17,18] and study the diagnostic [19,20] and prognostic value of these symptoms [21,22,23].

In the months that followed, it was possible to monitor the recovery of olfactory function over time, confirming that, in most cases, the loss of smell following viral infections recovers within a few weeks. However, reliable studies with follow-up at 6 [24,25,26], 12 [27,28,29,30] and 24 months [31,32] have been conducted, confirming that up to 5% of patients develop long-term disorders with significant effects on quality of life [33]. For the first time, it was possible to identify the risk factors [34,35,36,37] for the development of persistent olfactory disorders, including female gender [26,38], younger age [38,39], smokers [40] or non-smokers [38], hypertension [41], diabetes [41], depression [42], symptoms such as fever [38] and nasal obstruction [43], circulating levels of D-dimer during infection [44], and levels of nasal immunoglobulins [45,46].

At the same time, important new advances have been made in studying the pathogenesis of olfactory disorders after viral infections [47,48,49,50,51,52]. All of these efforts have ultimately led to numerous therapeutic trials to identify effective therapies for the prevention and treatment of persistent olfactory disorders [53,54,55,56,57,58].

This Special Issue represents a good summary of all of these research areas. The study by Haener et al. [59] shows that COVID-19-related olfactory disorders are significantly more frequent and severe than those caused by other cold viruses. The reviews by Scotto et al. [60] and Pang et al. [61] analyze the correlations between taste alterations and SARS-CoV-2 establishing the diagnostic value of this chemosensitive disorder. The diagnostic value of olfactory disorders is instead the subject of studies by Mazzatenta et al. [62,63] and Jungbauer et al. [64].

The two contributions by Schambeck et al. [65] and Albayay et al. [66] analyze the prevalence of long-term olfactory disorders, confirming the important impact of this problem, as underlined by a study of the impact on the quality of life of persistent olfactory disorders by Vaira et al. [67].

Callejon-Leblic et al. [68] and Tipirdamaz et al. [69], in two large studies of 421 and 354 patients with COVID-19, respectively, identified older age, cacosmia and asthma as a risk factor for developing a long-term olfactory disorder. Finally, Hintschich et al. [70] and Tsuchiya [71] analyzed the effects of nasal corticosteroids and zinc in the treatment of COVID-19-related olfactory and gustatory disorders.

In the last few years, we have learned much about post-viral chemosensory disorders. In July 2020, an article by Cooper et al. [72] ran with the title: “COVID-19 and the chemical senses: supporting players take center stage”. The management of chemosensory disorders has always represented a research niche that has suddenly ended up at the center of public attention. Today and in the future, this same public will increasingly demand solutions to their problems, even if not related to COVID-19. Alongside the great efforts made by researchers in recent years, it is necessary for health systems to recognize the importance of chemosensory disorders by providing those who have to deal with these patients adequate organizational and economic tools to cope with increasing demands for assistance.
